# Performance of Ultrasound Techniques and the Potential of Artificial Intelligence in the Evaluation of Hepatocellular Carcinoma and Non-Alcoholic Fatty Liver Disease

**DOI:** 10.3390/cancers13040790

**Published:** 2021-02-14

**Authors:** Monica Lupsor-Platon, Teodora Serban, Alexandra Iulia Silion, George Razvan Tirpe, Alexandru Tirpe, Mira Florea

**Affiliations:** 1 Medical Imaging Department, Regional Institute of Gastroenterology and Hepatology, Iuliu Hatieganu University of Medicine and Pharmacy, 400162 Cluj-Napoca, Romania; 2 Medical Imaging Department, Iuliu Hatieganu University of Medicine and Pharmacy, 400162 Cluj-Napoca, Romania; teodora.serban@elearn.umfcluj.ro (T.S.); alexandra.iuli.silion@elearn.umfcluj.ro (A.I.S.); 3 County Emergency Hospital Cluj-Napoca, 3-5 Clinicilor Street, 400000 Cluj-Napoca, Romania; razvantirpe@gmail.com; 4 Research Center for Functional Genomics, Biomedicine and Translational Medicine, Iuliu Hatieganu University of Medicine and Pharmacy, 23 Marinescu Street, 400337 Cluj-Napoca, Romania; altirpe@gmail.com; 5 Community Medicine Department, Iuliu Hatieganu University of Medicine and Pharmacy, 400001 Cluj-Napoca, Romania; miraflorea@umfcluj.ro

**Keywords:** hepatocellular carcinoma, non-alcoholic fatty liver disease, ultrasonography, contrast enhanced ultrasound, artificial intelligence, steatosis, focal liver lesion

## Abstract

**Simple Summary:**

The increasing prevalence of non-alcoholic fatty liver disease (NAFLD) represents a challenge for the current medical systems. If NAFLD is left undetected and untreated, it can progress towards fibrosis, cirrhosis, and hepatocellular carcinoma (HCC). To date, ultrasonography (US) is the first-line examination indicated to NAFLD patients that also screens for other focal liver lesions (FLLs). The downside of conventional B-mode US is that it cannot accurately quantify steatosis and fibrosis and cannot further characterize FLL certainly—is it cancer or is it not? Ultrasound contrast agents (UCAs) allowed physicians to further evaluate the FLL for the diagnosis of HCC. This review discusses the performance of US techniques in NAFLD and NAFLD-related HCC diagnosis, as well as of artificial intelligence (AI)-based methods, specifically the usefulness and assistance of deep learning algorithms for improving liver US image processing.

**Abstract:**

Global statistics show an increasing percentage of patients that develop non-alcoholic fatty liver disease (NAFLD) and NAFLD-related hepatocellular carcinoma (HCC), even in the absence of cirrhosis. In the present review, we analyzed the diagnostic performance of ultrasonography (US) in the non-invasive evaluation of NAFLD and NAFLD-related HCC, as well as possibilities of optimizing US diagnosis with the help of artificial intelligence (AI) assistance. To date, US is the first-line examination recommended in the screening of patients with clinical suspicion of NAFLD, as it is readily available and leads to a better disease-specific surveillance. However, the conventional US presents limitations that significantly hamper its applicability in quantifying NAFLD and accurately characterizing a given focal liver lesion (FLL). Ultrasound contrast agents (UCAs) are an essential add-on to the conventional B-mode US and to the Doppler US that further empower this method, allowing the evaluation of the enhancement properties and the vascular architecture of FLLs, in comparison to the background parenchyma. The current paper also explores the new universe of AI and the various implications of deep learning algorithms in the evaluation of NAFLD and NAFLD-related HCC through US methods, concluding that it could potentially be a game changer for patient care.

## 1. Introduction

Hepatocellular carcinoma (HCC), the fourth leading cause of cancer mortality worldwide and the fifth and ninth most commonly diagnosed cancer in men and women, respectively, has changed its landscape. The incidence of non-viral HCC is increasing, since obesity, metabolic syndrome (MetS), and type 2 diabetes mellitus are a true epidemic [1,2]. Non-alcoholic fatty liver disease (NAFLD), the hepatic manifestation of the MetS, has become one of the leading causes of morbidity and mortality globally, affecting approximately 25% of the world’s population [3,4]. NAFLD diagnosis requires identifying steatosis by imaging methods or histology, as well as the exclusion of significant alcohol consumption or other competing etiologies for fat accumulation [5,6,7,8]. The burden of NAFLD is especially important since it can progress towards fibrosis, cirrhosis, and NAFLD-related HCC [4]. Moreover, this pathology is recognized not only as the most common etiology of chronic liver disease, but as a major cause of cirrhosis and HCC, and it is expected to become the leading recommendation for liver transplantation in this decade [9,10]. Mittal et al. [11] reported that NAFLD individuals are fivefold more likely to develop HCC without underlying cirrhosis, compared to patients suffering from other chronic liver diseases. Notably, most NAFLD patients included in this study suffered from obesity and diabetes, supporting the pathogenetic hypothesis. The carcinogenesis process behind the HCC development in NAFLD is not completely understood, but different metabolic comorbidities, such as obesity and insulin resistance, are being incriminated, along with the pro-inflammatory status and the genetic predisposition identified in some patients [12]. It is well known that the presence and severity of fibrosis are important prognostic factors in NAFLD. Using combinations of non-invasive methods, such as composite scoring systems and/or transient elastography, enabled risk stratification of patients by fibrosis stage [13,14].

At present, ultrasonography (US) is the first-line imaging modality used for HCC screening among cirrhotic patients. Having a sensitivity of 40–81% and specificity of 80–100% for surveillance purposes, US is useful in cirrhotic and noncirrhotic patients, including NAFLD that should also undergo routine surveillance [15,16]. However, this technology encounters several limitations in the NAFLD population, considering that the body mass index (BMI) among these subjects is usually increased [17]. In addition, current guidelines lack specific recommendations for primary HCC prevention and, moreover, do not include clear recommendations for a cost-effective surveillance of the non-cirrhotic NAFLD patients carrying a risk of HCC development [14,18]. Surveillance among these individuals remains controversial since mass screening using conventional US has low cost-effectiveness [19]. Evidence of improved non-invasive diagnosis is in growing progress, requiring updating and reflection of data in clinical practice. In this review, we provide an updated analysis of the performance of ultrasound techniques and the potential contribution of artificial intelligence-based methods in the US evaluation of NAFLD/NASH and NAFLD-related HCC.

## 2. Conventional Ultrasonography

### 2.1. Evaluating NAFLD Using Conventional US

Currently, conventional US is recommended as the first-line examination for patients with high clinical suspicion of NAFLD, given the large number of advantages: It is cost effective, broadly available, non-invasive, appropriate for re-examination, and highly convenient for patients [5,6,7,20,21]. Ultrasound is sensitive (85%) and specific (95%) for detecting moderate to severe steatosis (>33% steatotic hepatocytes), but its sensitivity deteriorates when <30% of hepatocytes are affected [21,22]. Moreover, increased echogenicity, the main US finding in NAFLD patients, is present in fibrosis and early cirrhosis as well, reducing the reliability of US in coexisting liver disease etiologies [13].

#### 2.1.1. Ultrasound Diagnostic Criteria for Hepatic Steatosis

**Figure 1:**
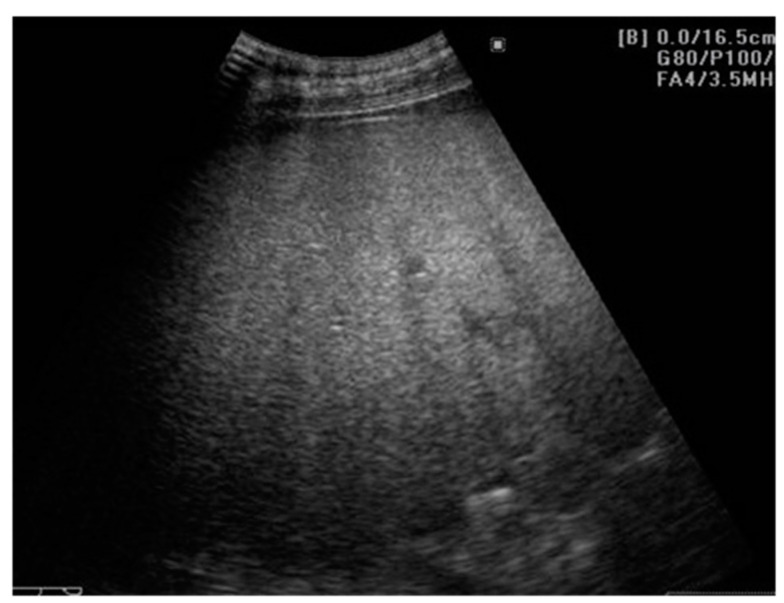
Hepatic steatosis. The 2D-US examination is showing an enlarged liver, with increased echogenicity and posterior beam attenuation, with a slightly inhomogeneous structure of fine granularity, without any FLLs. Even if this aspect is highly suggestive of hepatic steatosis, conventional US is unable to properly quantify the fatty amount of the liver. Also, 2D-US cannot specify whether fibrosis is present or not. Usually, steatosis and fibrosis coexist and therefore “steato-fibrosis” is the preferred term in this situation.

Besides hepatomegaly, the fat droplets interact with the ultrasound and a greater number of echoes return to transducer, displaying the well-known bright, hyperechoic liver, compared to the right kidney. Furthermore, the hyperechoic appearance of the liver results in a poor visualization of portal veins, liver capsule, and the gallbladder wall. In addition, lipids attenuate the ultrasound, leading to posterior darkness effect and a decreased visualization of the structures within the parenchyma and of the diaphragm, as seen in Figure 1. Also, altered liver hemodynamics detected with Doppler US can be seen, with one noteworthy example being the abnormal waveforms of the hepatic veins [20,21,23,24,25].

#### 2.1.2. Quantitative Assessment of Hepatic Steatosis

The hyperechoic image is a qualitative feature and is dependent on the subjective interpretation of the examiner, leading to variability in results and low reproducibility [26]. A grading system of steatosis has been proposed in attempt to reduce the observer bias, using the hepatic, periportal, and diaphragmatic echogenicity, as exemplified in Table 1. However, all the criteria used to grade steatosis remain subjective.

#### 2.1.3. US Performance for Steatosis Detection

Conventional US is an accurate and highly reliable diagnostic tool for steatosis assessment. Wang et al. [28] found that the agreement rate of US as compared to histology is 61.4% in assessing the steatosis severity and 74.3% in diagnosing steatosis. A meta-analysis by Hernaez et al. [22] estimated that the US sensitivity to detect moderate and severe steatosis confirmed by histology is 84.8% and the specificity is 93.6%. However, when mild steatosis is taken into consideration (fat content less than 20%), the traditional US has low sensitivity and therefore a high false negative rate of 55% [29]. The Dasarathy [29] prospective study observed that a combination of abnormal sonographic features increases the overall sensitivity and specificity. Another noteworthy remark was the fact that the sensitivity and specificity of hepatic vein blurring was higher than the one of the portal vein blurring, and there was a high concordance between hepatic vein blurring and the increased echogenicity. Therefore, the combination of portal vein blurring and liver brightness was a better sonographic predictor for hepatic steatosis [30].

Recently, a series of quantitative and semi-quantitative parameters have been implemented on US methods in order to overcome the limitation of low sensitivity in mild steatosis diagnosis. Some of these parameters exhibit a better performance than conventional US alone and have a better reproducibility and reliability [26,31,32,33]. Such parameters include attenuation (AC) and backscatter coefficients (BSC), the hepato-renal index (HRI) and ultrasound envelope statistic parametric imaging (known as speckle statistics). Hepatic steatosis correlates positively with these parameters. However, further studies are warranted to validate their potential widespread clinical use [31,34].

In addition, the controlled attenuation parameter (CAP) measured by the vibration controlled transient elastography (VCTE), a widely available device, has a powerful contribution in steatosis evaluation. The potential of US elastography is a subject of high interest in the non-invasive evaluation of NAFLD and NAFLD-related HCC, as previously exemplified in a recent paper from our group [14].

#### 2.1.4. Ultrasonographic Steatosis Patterns

In the fatty liver, there are a number of different steatosis patterns that might be observed: diffuse, multinodular, focal geographic, focal nodular, intralesional, perilesional, subcapsular, periportal, perivenular steatosis, and hypersteatosis. Frequently, focal fatty infiltration occurs geographically, but pseudo-tumoral aspects are also possible [35]. Another mass like appearance can be given by areas of normal echogenicity as compared with the background hyperechoic parenchyma, termed “focal fatty sparing” (FFS). FFS are commonly found in segments IV and V, near the left portal vein or adjacent to the falciform ligament and the gallbladder fossa [36]. Due to their nodular appearance, FFS can lead to confusion with other focal liver lesions (FLLs) that have pathological implications.

#### 2.1.5. Limitations of Ultrasonography in Steatosis Diagnosis

As previously mentioned, fibrosis is a key histologic feature that needs to be considered in NAFLD patients and especially in NASH subjects. Fibrosis is an important confounder for steatosis evaluation using US, as they both appear hyperechoic [37]. A study of 118 biopsy-proven NAFLD subjects found that US sensitivity for detecting moderate to severe histological steatosis was 100% among individuals with mild fibrosis on histology [37]. However, it decreased significantly to 77.8% in those with advanced fibrosis. In fact, several studies found no correlation between the US characteristics and the grade of inflammation, ballooning, and fibrosis identified on histology, making it challenging to distinguish between simple steatosis and progressive NASH [38,39]. Several studies proved that US was unable to discriminate between simple steatosis and NASH [29,40].

Another limitation of US is the low sensitivity in patients with morbid obesity (BMI > 40 kg/m^2^) [41]. The subcutaneous fat reduces the ability of conventional US to evaluate liver echogenicity, especially among patients with high risk for NAFLD development [42]. In addition, as above mentioned, the method is unable to establish with certainty the degree of fatty infiltration.

### 2.2. NAFLD-Related HCC: Could Conventional and Doppler US Differentiate between Focal Liver Lesions (FLLs)?

According to multiple guidelines, B-mode US is the primary imaging technique used for HCC screening in high-risk patients [19,43]. Although B-mode US cannot make a final diagnosis, the main goal is to detect any focal area differing from the background parenchyma. The method represents a milestone in selecting further management, being suggestive when a tumor displays signs of malignancy. Therefore, any suspicion of malignancy should be considered a positive result of the ultrasound exam, and further evaluation of the lesion will need to establish a specific diagnosis [44].

The US Liver Imaging Reporting and Data System (LI-RADS) score was established by the American College of Radiology (ACR) as an algorithm for the evaluation and management of FLLs in order to improve high-risk patient care [45]. According to the LI-RADS algorithm, the size threshold determines the further path to diagnosis. Lesions measuring less than 1 cm are difficult to assess with certainty, regardless of the imaging method. Nevertheless, they are usually benign and require follow-up at 3–4 months. If the tumor remains unchanged after 2 years of surveillance, malignancy is excluded [19,43,46]. For larger lesions, the US findings are inconsistent and an in-depth characterization is mandatory. Malignancy suspicion is raised by large focal lesions with heterogeneous echostructure and signs of parenchymal distortion—defined as ill-defined area of heterogeneity, refractive edge shadowing, and distortion of the normal internal hepatic architecture [47]. HCC of any size shows variable echogenicity, but as it develops, the tumor might undergo fatty metamorphosis, leading to a hyperechoic structure and a higher risk of being confused with a hemangioma [48]. Another important finding is a new thrombus identified in the liver venous system, more frequently within the portal veins, which can represent a bland thrombus or tumor and strongly indicates a diagnosis of HCC [45,49].

**Figure 2:**
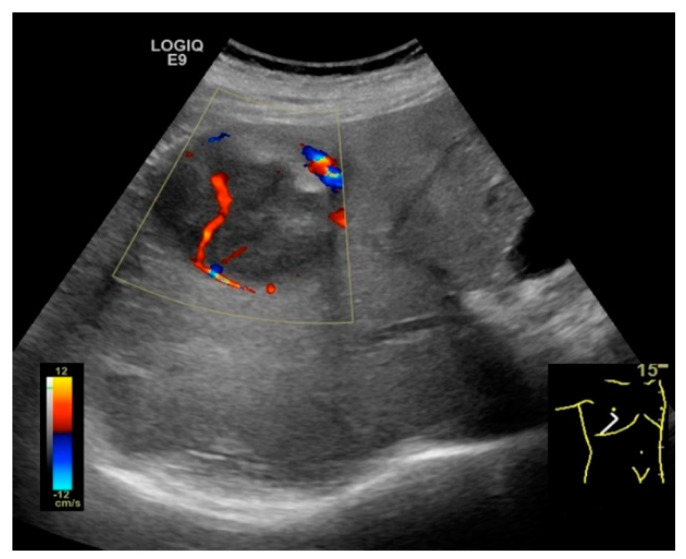
Hepatic steatosis. Focal liver lesion. 2D-US scan shows an enlarged liver with increased echogenicity and posterior beam attenuation. In addition, a focal parenchymal structure is observed in the right lobe. It is characterized as having decreased, heterogeneous echogenicity and internal vessels seen at Doppler examination. The diagnosis of the focal lesion remains uncertain and CEUS examination is necessary.

In addition, US Doppler assessment of blood flow and vascularization is useful, but not definitive (Figure 2) [48,50]. Central or peritumoral hypervascularity, basket pattern (vascular network at the periphery of tumor penetrating to the center) and the presence of pulsatile afferent flow signal with constant efferent flow are typical findings suggestive of HCC [50,51]. Moreover, spectral Doppler analysis offers parameters such as maximum flow velocity (Vmax) and pulsatility index (PI), which are useful in the differential diagnosis of several hepatic tumors. Notably, a very high value of the PI is suggestive for HCC, increasing the diagnostic efficacy of ultrasonic methods [47,52].

In NAFLD subjects, a notable confounder is represented by focal fatty sparing areas. However, lesions with typical location and shapes, as previously mentioned, and without mass effect, are suggestive ultrasonographic features for FFS [53]. Moreover, one study implied that FFS usually do not develop on previously homogeneous NAFLD, implying that a new ultrasonographic observation should be compared to former US examinations [54]. US Doppler comes as an adjuvant tool for conventional US. Usually, FFS are not hypervascular and do not distort the normal hepatic vessels. Nevertheless, the final diagnosis requires further imaging modalities, including contrast enhanced US (CEUS), computed tomography (CT), magnetic resonance imaging (MRI), or even US-guided biopsy [55].

Of note is a 2018 meta-analysis conducted by Tzartzeva et al. [56] that found an all stage pooled sensitivity for HCC detection of 84% with US, approximately equal to the CT/MRI sensitivities. In the same study, sensitivity for early-stage HCC was only 47% with US and 63% when combined with alpha-fetoprotein level (AFP), proving the limited use of US for small tumor detection. In addition, US findings are not specific, as appearances of FLLs overlap [48,57]. US allows accurate diagnosis for few FLLs, of which hemangioma, simple cyst, and calcifications are the most common [49]. In the same manner, US Doppler is limited in the diagnosis of focal nodular hyperplasia (FNH), in which the central artery traversing the central scar and its radial distribution are evocative in 80% of cases [53]. Concluding, most FLLs require a definitive characterization with a diagnostic multiphase contrast-enhanced examination (CEUS, CT or MRI).

## 3. Contrast-Enhanced Ultrasonography (CEUS): An Add-on to the Diagnostic Power of Ultrasonography in NAFLD-Related HCC

### 3.1. General and Technical Considerations

Contrast-enhanced ultrasonography (CEUS) is a particular US technique that overcame several drawbacks of both the conventional B-mode and Doppler ultrasound techniques by adding the intravenous administration of microbubble contrast agents [58,59]. When analyzing the liver, CEUS provides real-time recording and interpretation of the ultrasound contrast agent (UCA) flow through the parenchyma. Dynamic contrast-enhanced ultrasound (DCE-US) has made quantitative assessment possible and readily available by analyzing the time intensity curve (TIC) and facilitating measurement of the blood flow parameters [60].

Currently, there are four Food and Drug Administration (FDA)-approved UCAs available worldwide: SonoVue/Lumason (Bracco Suisse SA, Geneva, Switzerland), Definity/Luminity (Lantheus Medical Imaging, Inc., North Billerica, MA, USA), Optison, and Sonazoid (GE Healthcare AS, Oslo, Norway) [61]. These UCAs consist of biodegradable gas microbubbles, equal or smaller in size than red blood cells, stabilized in a phospholipid or albumin shell [58,59]. Because of their physical size, all UCAs act as blood pool agents, allowing the representation of both small and large vessels [62]. While SonoVue, Definity and Optison are purely intravascular agents, Sonazoid is phagocytosed by the hepatic reticuloendothelial cells (Kupffer cells). This leads to increased clearance from the vascular distribution volume and significant persistence in the liver, termed the post-vascular phase (also known as the Kupffer cell phase). Regardless of whether microbubbles are within the reticuloendothelial system or utterly within the blood pool, they can be easily destroyed by the ultrasound energy emitted by the examiner, providing real-time visualization of different vascular phases [60]. Given the dual blood supply of the liver, from the hepatic artery and the portal vein (25–30% and 70–75% of the total blood supply respectively), three different vascular phases have been defined: the arterial (AP), portal venous (PVP), and the late (LP) phase [61].

Moreover, UCAs enable the characterization of the vascular architecture through the phase-specific contrast enhancement in comparison to the background liver parenchyma. These characteristics are highly suggestive diagnostic features for various FLLs [60,61]. However, it is mandatory to perform a thorough B-mode and color Doppler US evaluation of the liver beforehand, considering that cysts and calcifications can be easily misinterpreted due to complete absence of enhancement. In addition, the assessment of the underlying parenchyma is paramount in order to ascertain whether cirrhosis is present or not, which can be a game changer [61].

#### Indications, Advantages and Limitations of CEUS Compared to Conventional US

Currently, clinical practice guidelines recommend abdominal US surveillance for malignancy every 6 months among cirrhotic patients [19,43]. However, the detection of small HCC nodules is difficult in subjects with liver cirrhosis, since they usually present a coarse parenchyma [63]. In addition, a high percentage of NAFLD-related HCC cases arise on non-cirrhotic liver [64]. As previously mentioned, steatosis and obesity independently impair US sensitivity in NAFLD patients [17]. CEUS improves the accuracy of B-mode US, by adding a new dimension to the equation—it evaluates the enhancement properties and vascular architecture of FLLs as compared to the background parenchyma [59]. In an experimental NASH rat model, Carvalho et al. [65] reported increased sensitivity and specificity (71% and 96%, respectively) after contrast administration, compared to Doppler US (29% and 71%, respectively). For integrative purposes, we decided to summarize the indications, advantages, and limitations of the conventional US compared to CEUS in Table 2.

### 3.2. Assessment of Fatty Liver Progression Using CEUS

Studies evaluating chronic liver diseases using CEUS are rather scarce. However, fat accumulation is the key factor that leads to vascular impairment and increased vascular resistance. Technologies such as CEUS can appraise hepatic microcirculation and quantify early changes in the parenchymal flow, before the onset of fibrosis [73,74]. There are several in vivo studies that evaluated fatty liver progression and HCC development using untargeted CEUS [65,75,76]. Of note is the Pandit study [75] that identified disease progression using vascular parameters, concluding that NASH liver parenchyma has the lowest blood flow. On the other hand, Tsujimoto et al. [76] evaluated Kupffer cells dynamic and phagocytic activity in a rat NASH model using Levovist and observed a reduced contrast effect in the liver. In humans, several studies using transit time parameters, such as the hepatic vein transit time (HVTT), evaluated fibrosis in different chronic liver diseases including NAFLD, chronic hepatitis B, and chronic hepatitis C. They observed earlier arrival time of contrast agents in the hepatic veins in severe fibrotic patients, compared to their healthy counterparts, and concluded that intrahepatic hemodynamic changes, such as shunts or liver arterialization, play an important role in these changes [77,78,79,80]. To summarize, Table 3 embodies an overview of different techniques for fatty liver assessment.

### 3.3. The Evaluation of FLLs, Including HCC, in NAFLD Patients Using CEUS

Over the years, international guidelines sought to elucidate the role of CEUS for FLLs evaluation. At first, CEUS was considered an inappropriate diagnostic tool for HCC surveillance, and more expensive technologies, such as Contrast Enhanced CT (CeCT) or Contrast Enhanced MRI (CeMRI), were preferred [83,84]. However, in the past years, additional evidence has been published for all UCAs and proved otherwise [61]. The DEGUM multicenter trial [85,86,87], together with the multicenter study of Sporea et al. [88], showed that CEUS possesses powerful capacity in differentiating between malignant versus benign FLLs, as exemplified in Table 4. CEUS sensitivity ranges from 80–94% for all size focal lesions and 55–76% for those ≤20 mm, and the specificity from 82–98% for all size liver nodules and 80–98% for those ≤20 mm, providing similar performance to CT and MRI for characterizing FLLs [86,88,89]. Having 63–76% sensitivity and 87–98% specificity, CT enables full cross-sectional evaluation of the liver and provides staging information. Gadolinium-enhanced MRI offers a better depiction of intrinsic tumor characteristics than CT with 67–82% sensitivity and 86–94% specificity. Moreover, Gadoxetate-enhanced MRI is very sensitive for early and small lesions (≤20 mm) with 90–93% sensitivity and 87–91% specificity, facilitating the differentiation of early HCCs from cirrhosis-associated benign nodules. Functional MRI techniques, including diffusion-weighted imaging, MRI with hepatobiliary contrast agents, perfusion imaging, and magnetic resonance elastography are promising in providing additional imaging features for tumor microvascular invasion and growth patterns, allowing preoperative prediction and prognosis [90]. Emerging as the most accurate and cost-effective imaging modality for FLLs characterization, CEUS is currently recommended as the first-line method for hepatic lesions evaluation, especially in patients with inconclusive CT or MRI findings, or among those with contraindications for these techniques [61]. In addition, recent analyses reported that Sonazoid CEUS surveillance might be a cost-effective method to increase expected survival time among at-risk subjects [67,91]. However, routine use of CEUS for HCC screening among patients at risk is currently not recommended and further research is needed to find ways to integrate such technologies into the healthcare surveillance strategies [61].

#### 3.3.1. Diagnostic Features of Hepatocellular Carcinoma on CEUS

**Figure 3:**
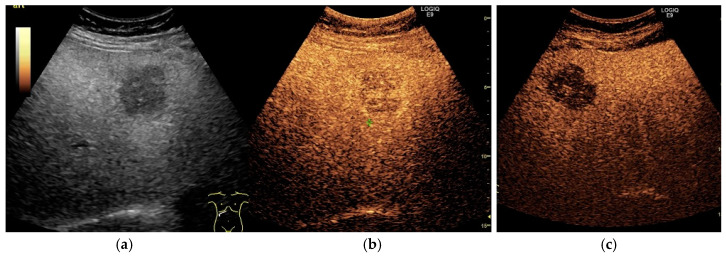
Hepatic steatosis. HCC. Conventional B-mode image (**a**), CEUS in the arterial phase (**b**), and CEUS in the late phase (**c**). The examination shows an enlarged liver with markedly increased echogenicity and a focal parenchymal lesion with decreased echogenicity (**a**). At CEUS technique, the lesion shows enhancement in the arterial phase (**b**), followed by washout in the late phase (**c**).

The major features for HCC diagnosis on CEUS are arterial phase hyperenhancement followed by mild washout with late onset in the portal and/or late phase, as depicted in Figure 3 [59,98]. However, studies demonstrated that the enhancement patterns largely depend on the degree of arterial vascularization and the differentiation grade of the tumor [99,100]. In regards to the vascularization, studies reported arterial phase hyperenhancement in 90–97.8% of HCC lesions while hypoenhanced observations were mainly well differentiated HCCs [87,98,100,101]. In spite of that, Von Herbay et al. [102] reported 28% non-hyperenhanced HCC nodules. These results might be explained by the inclusion of undifferentiated (G4) HCC nodules, considered by Yang et al. [98] as FLLs with decreased arterial supply. Regarding the diameter of the FLL, the Von Herbay study found a significantly higher incidence (95%) of hypervascularization in larger (>3 cm), well differentiated (G1) HCC lesions, in comparison to smaller (<3 cm) G1 HCCs (43%) [102].

Mechanistically, the relationship between washout patterns and cellular differentiation is influenced by the amount of portal veins in the suspected nodule. In the multistep hepatocarcinogenesis, the supplying vessels undergo major changes, with normal arteries and portal veins decreasing, while the abnormal neoplastic arteries increase [103]. Therefore, HCC with poorer grades of differentiation tend to present moderate washout, whereas well differentiated HCCs are likely to be iso-enhancing in the LP [99,100,104]. Another key feature for HCC evaluation consists of proper assessment of washout chronology. As a real-time imaging technique, CEUS enables precise assessment of washout onset, a fundamental characteristic for the CEUS LI-RADS classification. HCC typically shows washout with late onset (>60 s), while non-hepatocellular lesions, including intrahepatic cholangiocarcinoma (ICC), present early washout onset (<60 s) [59].

**Figure 4:**
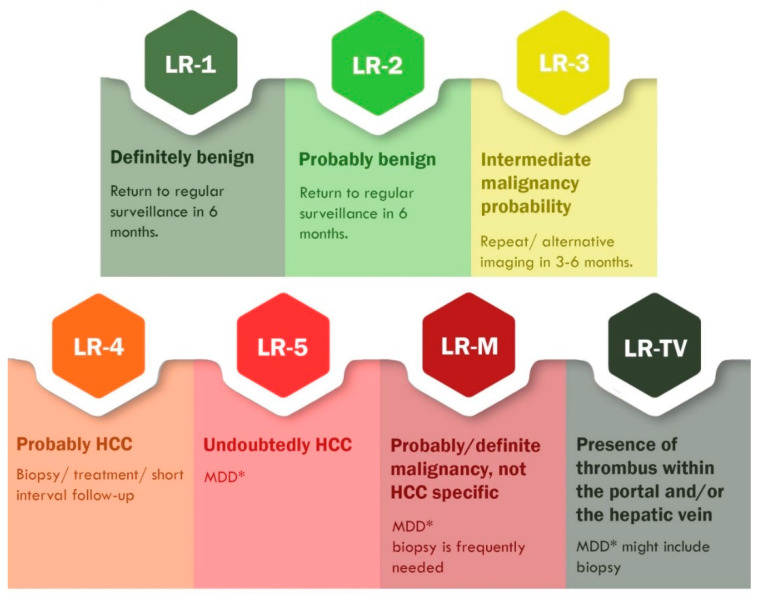
CEUS LI-RADS classification and management of FLLs according to the American College of Radiology [59,111]. This table includes only CEUS pure blood pool agents (SonoVue, Luminity). * MDD: multidisciplinary discussion; #MDD should be considered, since a recent prospective study found that 60% of CEUS LR-3 observations were HCCs [112].

The combined appraisal of the aforementioned diagnostic features makes it possible to evaluate the FLLs as malignant or benign in patients without underlying cirrhosis [87]. In those with liver cirrhosis and other risk factors for HCC development, CEUS can detect and characterize FLLs according to the LI-RADS classification. Released in 2016, CEUS LI-RADS is a standardized algorithm that classifies observations from LR-1 (a definitely benign lesion) to LR5 (an undoubtedly HCC). The LR score spectrum is illustrated in Figure 4. This table includes only CEUS pure blood pool agents (SonoVue, Luminity) [59,105,106,107,108]. A novel system proposed by Schellhaas et al. [109] redefined the population at risk for HCC, including patients with NASH and chronic hepatitis C with advanced fibrosis together with other key differences from the official CEUS LI-RADS. Nonetheless, their proposal was considered misleading by the ACR and consequently denied [110].

#### 3.3.2. HCC Particularities in NAFLD Patients

FLLs are frequent findings in clinical practice in patients with chronic liver diseases, such as cirrhosis or steatosis. One important aspect of HCC among NAFLD subjects remains its arduous detection. This is mainly due to subcutaneous fatty accumulation in addition to hepatic steatosis, which may alter the US visualization of small or early stage HCC nodules. Therefore, screening among these individuals remains controversial due to low cost-effectiveness. However, the recent study by Harris et al. [113] emphasized that screening among obese and NAFLD patients is of great interest and that clinicians should consider alternative imaging methods if US is limited.

**Figure 5:**
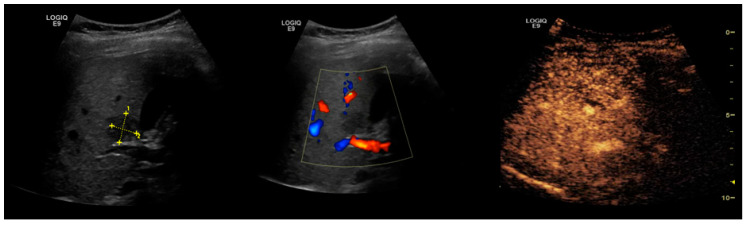
Focal sparing in the pericholecystic region. Hypoechoic image located around the gallbladder in a fatty liver. The Doppler examination cannot reveal any vessels within the lesion. On CEUS, the enhancement was homogeneous during the arterial, portal, and parenchymal phases, without any apparent focal lesions.

Another important aspect among NAFLD patients remains the wide variety of differential diagnosis. In particular, focal fatty changes, either by fat depositions or fatty sparing, may also occur, impairing the diagnostic accuracy of B-mode US examination. However, an iso-enhancing observation during all phases, without washout on CEUS, enables proper diagnosis, without the need for further imaging (Figure 5) [59,114]. Also, several studies aimed to elucidate whether underlying hepatic condition may alter lesions enhancement patterns. Yang et al. [98] reported no significant difference on the dynamic enhancement of HCC using CEUS in patients with or without cirrhosis.

#### 3.3.3. Sonazoid-Enhanced US—A Breakthrough in the CEUS Practice

As mentioned beforehand, Sonazoid accumulates in the reticuloendothelial system. Moreover, Sonazoid-enhanced US facilitates FLLs characterization, histological grading and guided percutaneous ablation therapy [115,116]. It is well-known that macrophages play an important role in malignancies [117]; Kupffer cells are specialized macrophages localized within the lumen of the liver sinusoid; the absence of these Kupffer cells in poorly differentiated HCCs usually causes contrast defect, corresponding to hypo-enhancement in the post vascular phase [58]. Nonetheless, in the Arita study [118], half of the well differentiated HCCs did not show lacking enhancement in the Kupffer cell phase. Recently, a meta-analysis by Wu et al. [119] found that Sonazoid has the highest diagnostic accuracy among all UCAs. However, being available only in Japan, South Korea, and Norway so far, only four studies were included in the Wu meta-analysis. Therefore, further worldwide research is needed in order to integrate Kupffer cell agents in the CEUS LI-RADS algorithm for FLLs characterization, considering the large palette of advantages that this method could bring to the clinician [105].

## 4. Artificial Intelligence in the Ultrasonographic Evaluation of NAFLD and NAFLD-Related HCC: A Potential Pillar

Ultrasound imaging is a widely used technique in today’s clinical practice. It provides both qualitative and quantitative information in a non-invasive manner, which benefits the patient. The classic examination performed by the radiologist/radiographer is operator-dependent, subjective, and cannot differentiate between steatosis and fibrosis; furthermore, conventional B-mode US is not able to establish the exact amount of fat accumulation in the hepatocytes. Artificial intelligence could revolutionize the evaluation of the images through a detailed and comprehensive analysis. Computerized image analysis can detect different textures from the ultrasound acquisitions, based on the physical and architectural alterations that affect the propagation of US waves. The use of computers in US image analysis started several years ago with the introduction of methods like grayscale analysis, ultrasound histogram, attenuation and/or texture information, and computer-assisted quantitative analysis of ultrasound beam echo amplitude [120,121,122]. Another method that can appraise steatosis severity is the computerized calculation of the hepatorenal ratio, with a sensibility of 91.3% and a specificity of 83% if the hepato-renal difference is ≥7 dB [30]. However, the efficacy of these rudimentary computerized methods remains questionable and the progress towards the applications of artificial intelligence is within our grasp.

In the past years, artificial intelligence (AI)-based methods, especially deep learning (DL) algorithms, gained extensive attention in the field of ultrasound imaging. In broad terms, we highlight two AI techniques with applications within the imaging field—machine learning (ML)-based algorithms with its more advanced class of DL. Convolutional neural networks (CNNs) are the most popular DL architecture used in medical imaging, although they require large amounts of training data [123,124]. AI-based algorithms that guide the examiner towards the best image acquiring position have been developed, so that, in the future, the examiner will not necessarily need to have previous US technique knowledge. In the near future, DL algorithms may provide accurate interpretation of various US images acquired by the examiner while returning a probable imaging diagnosis [125], assisting the physician in completing the clinical diagnosis and prioritizing urgent cases based on entities identified in the scans [126]. This section aims to discuss the current state of the AI research in the US evaluation of NAFLD and NAFLD-related HCC, focusing on the clinical applications of AI-based methods rather than the technology behind it.

Radiomics emerged as a new method in improving the accuracy of the clinical decision making based on medical imaging reports. It refers to the high-throughput mining of data from medical imaging. In general terms, the workflow of radiomics begins with the first step referring to the acquisition of standardized images, followed by the segmentation of the entities present within the image (either automatically, or by the physician) in order to define the desired region of exploration. Next, quantitative features, such as intensity levels, texture pattern, shapes, and the spatial interrelation of different entities are retrieved from the selected region with a consequent analysis based on complex algorithms. The most prominent data are investigated in relationship with treatment and prognosis, the main goal being accurate risk stratification [127,128,129].

From a brief technical perspective, the goal of the machine learning (ML) techniques is to study the underlying US features and transform them into information for segmentation or classification [130]. Furthermore, ML methods can be supervised and unsupervised; in supervised ML algorithms, the classifier is trained on an existing database containing US images that are labeled with the required outputs. Contrarily, unsupervised learning algorithms identify similarities in the input data, with no labels provided [131].

DL methods are a subclass of machine learning algorithms in computer science [130]. In the learning phase of DL algorithms, the labeled US images are randomly divided into two separate groups—training and validation; images from within the training group are used to automatically identify features and a specific model learned. Next, the validation group is used in order to estimate the performance of the best learned model identified. The DL algorithm will then be able to apply the learned algorithm to analyze a new US image and to make predictions. [131]. CNNs are the most popular DL architecture used in medical imaging and are inspired from the biologic neural networks, containing multiple computational units entitled artificial neurons that analyze the input images [132].

An unsupervised neural network that is worth mentioning is represented by stacked autoencoders. Briefly, these algorithms learns the representation of the input data by attempting to reconstruct it [130].

### 4.1. The Applications of AI in the Ultrasonographic Evaluation of NAFLD

As mentioned beforehand, the gold standard for NAFLD diagnosis is biopsy, but its invasiveness severely limits the method to specialized healthcare units. Currently, US is an invaluable non-invasive technique in the first-line examination of patients with clinical suspicion of NAFLD. Although a number of studies have tried to standardize a grading system for the US evaluation of NAFLD, all criteria still remain subjective. Considering that the Hernaez meta-analysis found a 84.8% sensitivity and a 93.6% specificity for detection of moderate and severe steatosis, whilst mild steatosis had considerably lower sensitivity [22], we sought to determine whether AI could potentially improve the US detectability of NAFLD.

A study by Han et al. [133] sought to evaluate DL algorithms that use radiofrequency (RF) data for NAFLD evaluation, analyzing 204 participants with 140 NAFLD-affected patients. Reference was set with MRI-derived proton density fat fraction (PDFF). Two one-dimensional CNN algorithms were developed—a binary classifier and a fat fraction estimator. Furthermore, the Han study divided participants into training group (*n* = 102) and test group (*n* = 102) by stratified randomization. The CNN algorithms were then developed through cross-validation, using the training group, and further evaluated in the test group. The Han study showed a high classification accuracy classifier (96%) with an AUROC of 0.98. Moreover, the sensitivity in the RF without time gain compensation was 97% [95% CI: 90–100%] and specificity 94% [95% CI: 79–99%] [133].

Furthermore, in a study by Biswas et al. [134] that included 63 patients, the accuracy, sensitivity and specificity for detecting fatty liver disease and making the risk stratification based on deep learning ultrasound (US) was 100%. The AUROC of deep learning method was 1.0 compared to extreme learning machine, which had an AUROC of 0.9222, sensitivity of 93.33% and specificity of 90.83%; support vector machines (SVM) had an AUROC of 0.8208, average sensitivity of 64.21%, and specificity of 93.56%, highlighting the better performances of deep learning technology in this study. Another study by Byra et al. [135] proposed the use of a CNN model for liver steatosis assessment in B-mode ultrasound imaging. Their work included 55 severe obese patients, 38 of whom had fatty liver disease. The overall result was significantly better than conventional B-mode US, with an AUROC of 0.977, accuracy of 96.3%, sensitivity of 100% and specificity of 88.2%, whereas the accuracy when using the hepatorenal index was 90.9% and the accuracy of gray-level co-occurrence algorithm was 85.4%. The Cao study [136] recruited 240 patients from routine abdominal US examinations and compared the performance of envelope signal value, gray scale value, and deep learning in diagnosing NAFLD, as well as differentiating between mild, moderate, and severe steatosis. The DL index did not follow a Gaussian distribution, but presented obvious differences between the mild, moderate, and severe NAFLD groups. In the Cao study, in terms of diagnosing NAFLD, deep learning-based algorithm had the best performance, with the highest DL index and an AUROC of 0.933, compared to gray scale value (AUROC = 0.857) and envelope signal (AUROC = 0.859). Whilst all three methods showed poor diagnostic capability in terms of NAFLD scoring between mild and moderate NAFLD (AUROC < 0.7), DL index showed much better capability (AUROC = 0.958) in distinguishing between moderate and severe NAFLD [136].

As such, the existing data within the literature suggests that deep learning methods can be a viable addition to the current clinical practice in diagnosing NAFLD. However, further studies are required in order to standardize this approach.

### 4.2. The Applications of AI in the Ultrasonographic Evaluation of NAFLD-Related HCC

The clinical day-to-day life proved that there is a need to detect HCC in early stages, but the current limitations regarding imaging techniques hamper the early diagnosis of this malignancy. AI is constantly and steadily evolving and could become an important player in early detection and staging. For comprehensibility purposes, Table 5 presents a brief overview upon the latest studies involving the potential of AI in the US detection of HCC and NAFLD-related HCC.

Taking into consideration all the aforementioned studies, our opinion is that AI can dramatically improve the US detection of NAFLD and NAFLD-associated pathological entities, such as cirrhosis and HCC, through DL algorithms.

### 4.3. Advantages and Pitfalls of Future AI-Based Solutions

AI techniques present a number of advantages when they are employed. The diagnostic accuracy of a number of pathologies can greatly increase, which can be translated into a better patient survival in the case of early HCC detection. Our paper has identified recent studies that demonstrate the wide potential of DL algorithms in identifying NAFLD and NAFLD-related HCC [141]. We also highlight the increase in productivity and the improvement in the clinical decision making, which essentially translates in better patient satisfaction.

However, there are different elements that can hinder the development of ML solutions and currently limit their applicability in US medical imaging. In order to achieve good learning performance with deep learning, there is a need for large sets of data during the training process from which the algorithms “learn”, which may currently be unavailable. For the supervised methods, the experts would have to swift through massive datasets and manually add annotations for the specific task, posing a real question of how to train such a model in a cost-effective and time-effective manner. Furthermore, there are several US-related features that can hamper the learning power of the AI: the artifacts in B-mode liver ultrasound (e.g., acoustic enhancement, comet tail, mirror image) or in Doppler mode (such as aliasing phenomenon or blooming artifact) must be recognized and not mistakenly diagnosed by the computer as pathologic. On the other hand, the US parameters (gain-brightness, depth, TGC curve, field of view) and the selection of probes must also be taken into consideration. Therefore, because of the variables related to the US machine as well as to the patient, the need for standardization is vital in order to achieve good results.

Eventually, there are potential biases that need to be considered before integrating AI in clinical practice. In the “anchoring effect”, the operator tends to make an interpretation in relation with the initial reference and can get biased in the decision making [142]. The second bias is one that could occur in systems based on supervised learning and is called the “bandwagon effect”. Knowing that the automated decision is based on a large collection of annotations, the operators tend to position their opinion with the algorithm [143].

Even if the medical imaging could benefit from the developments in computer science, mainly CNN, there is still a need to standardize the evaluator potential in order to achieve the best possible results. The constant evolution of the automated systems shines a bright light towards the future of ultrasound imaging.

## 5. Concluding Remarks

The silent progression of NAFLD towards NAFLD-related HCC prompts for accurate disease-specific surveillance tools that present a high accuracy. Ultrasound-based methods are currently the epicenter of NAFLD evaluation, with B-mode US being the first-line examination in high clinical NAFLD suspicion patients.

US-based methods are a powerful addition to the clinical examination in NAFLD patients, providing qualitative, quantitative, or both qualitative–quantitative information in NAFLD, depending on the technique used. Conventional B-mode US is a broadly available, cost-effective, non-invasive method that returns only qualitative-subjective information and has a reported sensitivity of 85% and 95% specificity for detecting moderate to severe steatosis, but lacks accuracy in the evaluation of mild steatosis. Furthermore, conventional B-mode US can identify focal liver lesions, but cannot make an in-depth characterization; malignancy is suspected in large focal lesions with heterogeneous echostructure and signs of parenchymal distortion. Doppler blood flow evaluation can identify a central or peritumoral hypervascularity, basket pattern, or the presence of pulsatile afferent flow signal with a concomitant constant efferent flow, which are suggestive of HCC but not definitive. Although these US techniques are not the first-line HCC diagnostic methods, they remain important first-line screening and surveillance tools. In regard to CEUS, the add-on of UCAs has rendered possible the further characterization of FLLs by adding a new real-time quantitative assessment into the equation. As an accurate and cost-effective imaging modality for hepatic lesion evaluation, CEUS provides the HCC diagnosis through the standardized LI-RADS score and the characteristic arterial phase hyperenhancement followed by mild washout with late onset in the portal/late phase. The use of the current UCAs and US contrast-specific techniques has brought CEUS to a similar performance to CT and MRI for the characterizing focal liver lesions. In addition, compared to dynamic CT and MRI, US can be performed in real time, is less expensive, and has no associated nephrotoxicity or ionizing radiation.

The current paper also underlines the wide potential of Artificial Intelligence-based methods, with a focus on deep learning algorithms, in the NAFLD and NAFLD-related HCC’s US images analysis. The literature search has identified a number of studies focused on NAFLD and NAFLD-related HCC that prove an increase in the diagnostic accuracy of these methods, when deep learning methods are employed. Our opinion is that AI could potentially be a game changer that widens the power of US based methods and, finally, benefits the patient by the early detection of NAFLD-related HCC.

## Figures and Tables

**Table 1:** Criteria used in the B-mode US grading of steatosis [5,27].

Grade	Ultrasonographic Features
I: mild steatosis	Liver echogenicity slightly increased and normal visualization of portal vein wall and the diaphragmatic outline.
II: moderate steatosis	Liver echogenicity moderately increased with slightly impaired visualization of portal vein wall and diaphragmatic outline.
III: severe steatosis	Liver echogenicity markedly increased with poor or no visualization of portal vein wall, diaphragmatic outline, and posterior portion of the right hepatic lobe.

**Table 2:** Indications, advantages, and limitations of CEUS as compared to B-mode and Doppler US.

	Conventional B-Mode and Doppler Ultrasound	Contrast Enhanced Ultrasound (CEUS)
Indications	HCC surveillance for high risk patients [19,43] Guides biopsy or treatment [66]	Evaluates nodules ≥ 10 mm observed at US surveillance [59] Guides biopsy or treatment for observations that are undetectable or inconspicuous on US [59,61] Selects the most relevant lesion/lesion component for biopsy [59,61] Evaluates lesions with inconclusive histology [59] Better characterization of arterial phase enhancement in inconclusive CT/MRI [59] Differentiates between benign and malignant portal vein thrombosis [59,61] First line contrast imaging modality in patients with renal insufficiency [61]
Advantages	Broadly available [20] Free from ionizing radiation [21] Cost-effective Non-invasive Typical HCC features of Doppler findings are available [50] The wide variety of Doppler methods (color/spectral/power Doppler) for better assessment of FLLs [47,67]	UCAs are safe in adult and pediatric individuals [61] The possibility of re-administration of UCAs for better assessment of suspicious observations [61] Avoids unnecessary further imaging for benign lesions [59] Absence of ionizing radiation [68] Real-time and quantitative assessment [61] Cost-effective [69,70] Excludes pseudovascular lesions detected on CT or MRI such as arterioportal shunts [71]
Limitations	Low sensitivity in patients with morbid obesity [41] Steatosis leads to acoustic beam attenuation [36,72] Unable to differentiate between simple steatosis and progressive NASH [38,39] Unable to differentiate between steatosis and fibrosis [37] Inadequate to assess with certainty the degree of fatty infiltration [30] Focal fatty deposition or sparing areas can lead to confusion with other FLLs [35] Low sensitivity for early-stage HCC [56] Overlap of FLL appearance on the US image [57]	Unsuitable for HCC staging [60] Subdiaphragmatic or deep lesions are difficult to reach and characterize properly [59] Limited penetration in obese patients [59] Severe hepatic steatosis alters signal transmission through the parenchyma [59]

**Table 3:** Characteristic of NAFLD by different non-invasive methods.

Technique	Features
B-mode US	Hepatomegaly Bright, hyperechoic liver compared to the right kidney Posterior beam attenuation Difficult visualization of echogenic structure, such as the portal vein wall, the gallbladder, the diaphragm etc.
Doppler US	Abnormal waveforms of the hepatic veins (normal triphasic pattern disappears) [81] Velocity of the portal flow (flow peak maximum velocity and mean flow velocity) and the portal vein pulsatility index (VPI) are significantly lower in patients with fatty liver when compared to the controls; it also corelates with the severity of the fatty liver [82]
US elastography	Fibrosis assessment by means of hepatic stiffness measurement Steatosis evaluation by the instrumentality of the Controlled Attenuation Parameter (CAP) [14]
CEUS	Earlier arrival time of contrast agents in hepatic veins using the hepatic vein transit time (HVTT) Reduced contrast effect in the Kupffer cell phase

**Table 4:** CEUS capacity using different contrast agents (SonoVue, Sonazoid, Levovist) for differentiating between malignant versus benign FLLs.

Study	UCA Used	AUROC	Malignant Lesions				Benign Lesions					
			HCC		Metastases		Hemangioma		FNH		Hepatocellular Adenoma	
			Se (%)	Sp (%)	Se (%)	Sp (%)	Se (%)	Sp (%)	Se (%)	Sp (%)	Se (%)	Sp (%)
Auer et al. [92]	SonoVue	0.951	100	100	90	100	99	100	100	100	66.6	100
			(*n* = 7)		(*n* = 31)		(*n* = 74)		(*n* = 19)		(*n* = 3)	
Sawatzki et al. [93] ^1^	SonoVue	N/S	Se = 96–97.2 ^#^				Sp = 84.2–90.6 ^#^					
			(*n* = 37)				(*n* = 75)					
Zhang et al. [94] *	SonoVue Sonazoid Levovist	0.94	85	91	N/S		N/S					
			(N/S)									
Yue et al. [95] ^2^	SonoVue	0.70	Se = 72; Sp = 84.6									
			(*n* = 30)		(*n* = 30)							
Deng et al. [96] *	Sonazoid Levovist	0.93	86	87	N/S							
			(*n* = 30–104)									
Sporea et al. [88]	SonoVue	N/S	81.2	94.2	93.1	94.1	90.2	97.6	94.7	98.4	N/S	
			(*n* = 209)		(*n* = 109)		(*n* = 102)		(*n* = 19)		(*n* = 7)	
Friedrich-Rust et al. [68] *	SonoVue Sonazoid Levovist	N/S	88	N/S	91	N/S	86	N/S	88	N/S	N/S	
			(*n* = 2238)		(*n* = 1775)		(*n* = 1191)		(*n* = 602)		(*n* = 84)	
Xie et al. [97] *	SonoVue Levovist	0.9555	N/S				N/S					
Strobel et al. [86]	SonoVue	N/S	Se = 93.5 ^#^				Sp = 66.7 ^#^					
			(*n* = 154)				(*n* = 87)					
Seitz et al. [85] **	SonoVue	N/S	86.1	96.6	93.6	82.4	62.5	97.3	57.1	99.3	N/S	
			(*n* = 7/40 **)		(*n* = 7/56 **)		(*n* = 48/9 **)		(*n* = 31/14 **)			

* meta-analysis, ** The Seitz study used two subgroups defined as subgroup A—without histological verification/subgroup B—with histological verification, *n* = number of patients taken into consideration, N/S = not specified, ^#^ these studies evaluate CEUS ability to identify malignant FLLs from benign ones without further classification; ^1^ This study included 2 NAFLD/NASH patients and 32 cirrhotic patients, but did not use the CEUS LI-RADS algorithm; ^2^ This study used parametric imaging CEUS, differentiating between HCC and metastatic liver cancer using quantitative parameters.

**Table 5:** A brief overview upon a selection of recent studies that evaluate the potential of AI techniques in the detection of HCC and NAFLD-related HCC.

Study	AI Technical Considerations	Accuracy of the AI Method
Bharti et al. [137]	Deep learning	Detection of Normal liver (*n* = 24 patients): Se/Sp = 96.3%/99.2% CLD (*n* = 25 patients): Se/Sp = 95.5%/98.0% Cirrhotic liver (*n* = 25 patients): Se/Sp = 97.5%/98.2% HCC on a cirrhotic liver (*n* = 20 patients): Se/Sp = 96.9%/99.8%
Hassan et al. [138]	Deep learning	Detection of HCCs, liver cysts and hemangiomas Se/Sp for the classification performance: 98.0%/95.7% Overall accuracy: 97.2%
Sato et al. [139]	Machine learning	Prediction of HCC (*n* = 539 patients with HCC, *n* = 1043 patients without HCC) Se/Sp = 93.27%/75.93% AUC = 0.940
Schmauch et al. [140]	Deep learning	Detection and characterization of FLL (benign vs. malignant) Training (*n* = 367 patients): Detection AUROC = 0.935 Characterization AUROC = 0.916 Test (*n* = 177 patients): AUROC = 0.891 for 7 different tasks
